# Exploratory Characterization of Grape Juice Fortified with *Vitis vinifera* Leaf Extract: A Proof-of-Concept Study

**DOI:** 10.3390/molecules31193502

**Published:** 2026-10-01

**Authors:** Cristina Matarazzo, Rosaria Cozzolino, Alessandra Fratianni, Livia Malorni, Tiziana Di Renzo, Massimo Di Renzo, Massimo Iorizzo

**Affiliations:** 1 Department of Agriculture, Environmental and Food Sciences, University of Molise, 86100 Campobasso, Italy; c.matarazzo3@studenti.unimol.it (C.M.); fratianni@unimol.it (A.F.); massimo.direnzo@unimol.it (M.D.R.); 2 Institute of Food Science, National Council of Research (ISA-CNR), Via Roma 64, 83100 Avellino, Italy; livia.malorni@isa.cnr.it (L.M.); tiziana.direnzo@isa.cnr.it (T.D.R.)

**Keywords:** grape juice, grapevine leaves, tocols, carotenoids, polyphenols, antioxidant activity, volatile organic compounds

## Abstract

The increasing demand for sustainable and phytochemically enriched foods has encouraged the valorization of agricultural by-products as ingredients for beverage formulation. This study investigated the enrichment of grape juice with *Vitis vinifera* leaf extract and characterized the resulting phytochemical, antioxidant, volatile, and sensory profiles. The fortified juice was compared with non-enriched grape juice and a commercial sample. Samples were analyzed at production (t_0_) and after 30 days of refrigerated storage (4 °C) for polyphenols, flavonoids, anthocyanins, carotenoids, tocopherols, and antioxidant capacity (ABTS, DPPH, and CUPRAC assays). Volatile organic compounds (VOCs), sensory properties, and microbiological stability were also evaluated. The fortified preparation showed higher concentrations of polyphenols, flavonoids, and anthocyanins than the non-fortified preparation. α-Tocopherol, γ-tocopherol, β-carotene, and lutein were detected exclusively in fortified samples. ABTS and CUPRAC assays showed comparatively high antioxidant activity values in the fortified preparation. Lower concentrations of anthocyanins, carotenoids, and tocopherols were observed at t_30_ than at t_0_, although substantial levels remained detectable after 30 days. HS-SPME/GC-MS detected 63 VOCs, with the fortified beverage showing a distinct volatile profile after 30 days of refrigerated storage, including comparatively high analytical responses for several monoterpenes and C6 alcohols. Sensory analysis revealed a distinctive profile characterized by greater sourness, bitterness, astringency, density, and aromatic complexity, together with lower overall sensory quality. Microbial counts remained below the respective detection limits under the investigated conditions. Overall, the investigated fortified preparation was characterized by a phytochemically enriched profile and by differences in antioxidant capacity, volatile composition, and sensory characteristics compared with the non-fortified preparation. These findings provide preliminary evidence of the compositional potential of grapevine leaf extract as an ingredient for phytochemically enriched grape-based beverages, while also identifying sensory constraints requiring formulation optimization.

## 1. Introduction

In recent years, food consumption trends have undergone a noticeable shift, with consumer choices increasingly focusing on nutritional value, health benefits, food safety, and the environmental impact of food production [1]. This shift has contributed to growing interest in enriched and fortified food products, particularly those incorporating ingredients with specific nutritional or phytochemical characteristics [2,3,4]. The popularity of plant-based beverages has grown significantly in recent years, encompassing a wide range of products based on soy, almonds, oats, rice, and fruits [5,6,7,8]. Owing to their diverse nutrient composition, these beverages may offer several nutritionally relevant properties [9] while providing consumers with options aligned with their dietary preferences and needs [10].

The cultivation of grapes represents a major agricultural activity worldwide, with wine production being its primary economic driver. Although traditional wine markets face several challenges, the low- and alcohol-free beverage sector has experienced substantial growth, largely driven by increasing demand from health-conscious consumers [11,12]. This expanding market presents significant opportunities for winemakers to increase sales, mitigate oversupply issues, and reach a broader and more diverse consumer base [13].

Agricultural activities generate considerable quantities of by-products that were historically regarded as waste. However, these by-products are now increasingly recognised as valuable resources due to their high content of bioactive compounds and bioactive components with potential applications across multiple areas [14].

In line with the objectives of the United Nations 2030 Agenda for Sustainable Development Goals (SDGs) [15], exploring the potential of agricultural by-products offers an opportunity to enhance agricultural sustainability and promote healthier food systems through the development of innovative, nutrient-rich ingredients while simultaneously reducing waste [16,17,18,19].

Throughout history, grapevine and winemaking by-products have played a significant role in Mediterranean, Greek, and Roman traditions, serving as natural ingredients, preservatives, and herbal remedies. Their nutritional characteristics and potential biological properties are largely attributed to their rich phytochemical composition [20,21,22,23,24]. Among these by-products, grapevine leaves remain largely underutilized despite their abundance and high content of bioactive compounds with antioxidant activity [25,26,27,28,29,30,31].

Previous studies have demonstrated that *Vitis vinifera* leaves are rich in bioactive compounds, including carotenoids, tocopherols, phytosterols, and polyphenols [27,28,32,33,34,35], with concentrations comparable to those reported in other grapevine tissues, including grapes and seeds [36,37,38,39,40,41]. Previous studies have demonstrated that grapevine leaves are a rich source of bioactive compounds and have characterized the phytochemical composition and biological activities of grapevine leaf extracts [42]. Other studies have explored the incorporation of grapevine-derived bioactive materials into fruit- and grape-based beverages [43]. However, to the best of our knowledge, the direct fortification of grape juice with a characterized grapevine leaf extract and the integrated assessment of its phytochemical, antioxidant, volatile, sensory, and microbiological characteristics have not been previously investigated. In particular, limited information is available on the simultaneous characterization of phytochemical composition, antioxidant capacity, volatile profile, and sensory characteristics in grape juices incorporating grapevine leaf extracts, as well as on the behavior of selected bioactive compounds during refrigerated storage. An integrated evaluation of these aspects is particularly relevant because phytochemical enrichment should be considered together with its potential implications for aroma, sensory quality, and the retention of bioactive compounds during storage.

Importantly, the incorporation of grapevine leaves into juice formulations may represent a potential strategy for obtaining phytochemically enriched grape-based beverages while supporting the valorization of this underutilized grapevine by-product [44].

Beyond their phenolic fraction, grapevine leaves contain lipophilic phytochemicals, including tocopherols and carotenoids, that may complement the phytochemical composition of grape juice. Thus, their potential value as a fortifying ingredient may lie not only in modifying global antioxidant-related indices but also in broadening the compositional profile of the beverage.

Within this exploratory framework, the contribution of the study lies in providing an integrated preliminary characterization of the compositional, antioxidant, volatile, sensory, and microbiological features of a grapevine-leaf-fortified grape juice formulation, while identifying potential formulation constraints that require further investigation. Based on these considerations, we hypothesized that incorporation of grapevine leaf extract into grape juice would be associated with a phytochemically enriched composition and with differences in antioxidant capacity, volatile composition, and sensory characteristics compared with the corresponding non-fortified preparation. Accordingly, the aim of the present proof-of-concept study was to prepare and exploratively characterize a grape juice formulation fortified with *Vitis vinifera* leaf extract. The investigated preparations were characterized for phytochemical composition, in vitro antioxidant capacity, volatile profile, sensory characteristics, and microbiological quality. For selected phytochemical and antioxidant parameters, measurements were obtained immediately after preparation (t_0_) and after 30 days of refrigerated storage (t_30_) to descriptively document changes observed within the investigated preparations over this period. VOC and sensory analyses were performed at t_30_ to characterize the beverages at that experimental time point. This exploratory approach was intended to provide a preliminary integrated characterization of the formulation without inferring generalized fortification, storage, or fortification × storage effects.

## 2. Results and Discussions

### 2.1. Microbiological Stability

Microbiological analyses showed that mesophilic aerobic bacteria, psychrotrophic bacteria, lactic acid bacteria, and Enterobacteriaceae were below the respective detection limit of 1 CFU/mL in all analyzed samples at both t_0_ and after 30 days of refrigerated storage. Acetic acid bacteria and yeasts and moulds were also below their respective detection limit of 10 CFU/mL at both sampling times. The absence of detectable microbial proliferation is consistent with the initial thermal treatment of the grape juice, refrigerated storage, and the naturally low pH of the beverage (<3.5), all of which provide conditions unfavorable to the growth of many spoilage microorganisms. Overall, these results demonstrate that no microbial proliferation above the detection limits of the applied methods occurred in the investigated beverages during the 30-day refrigerated storage period.

### 2.2. Phytochemical Composition and Antioxidant Activity

The investigated fortified grape juice (GJF) showed a different phytochemical and antioxidant profile compared with the non-fortified grape juice (GJ) and the commercial reference (CGJ) (Table 1). The enriched sample analyzed immediately after preparation (GJF_0_) exhibited the highest concentrations of total polyphenols (8.07 ± 0.13 g GAE/L), flavonoids (4.03 ± 0.09 g RtE/L), and anthocyanins (115.69 ± 2.35 mg MGE/L), corresponding to increases of approximately 13%, 31%, and 78%, respectively, compared with the non-enriched control (GJ_0_). These descriptive differences are consistent with a contribution of grapevine leaf extract to the overall phenolic profile of the investigated fortified preparation. In contrast, control samples showed lower levels of anthocyanins (65.10 ± 1.40 mg MGE/L), flavonoids (3.07 ± 0.07 g RtE/L), and total polyphenols (7.14 ± 0.12 g GAE/L), while the commercial sample (CGJ) displayed the lowest phenolic composition, particularly regarding total polyphenols (3.97 ± 0.07 g GAE/L) and flavonoids (1.73 ± 0.04 g RtE/L). After 30 days of storage at 4 °C, the enriched sample (GJF_30_) retained relatively high concentrations of total polyphenols (7.74 ± 0.11 g GAE/L) and flavonoids (3.70 ± 0.08 g RtE/L), whereas anthocyanin content decreased from 115.69 ± 2.35 to 80.51 ± 1.95 mg MGE/L after 30 days of refrigerated storage (Table 1). This reduction is consistent with the well-documented instability of anthocyanins during processing and storage, as their degradation is strongly influenced by several factors, including temperature, oxygen exposure, pH, enzymatic activity, copigments, sugars, metal ions, and degradation by-products, generally following first-order degradation kinetics [45]. Conversely, flavonoids are generally more stable in juice matrices, particularly under refrigerated conditions and during moderate storage periods, mainly due to the higher stability of glycosylated forms and their lower susceptibility to oxidative degradation [46]. Lower antioxidant activity values were observed at t_30_ than at t_0_ for some of the investigated preparations and assays, which may be associated with the partial degradation of phenolic compounds and antioxidant vitamins during refrigerated preservation [47].

The differences observed in phytochemical composition were closely reflected in the antioxidant activity measured by the ABTS, DPPH, and CUPRAC assays (Table 1), although each method exhibited distinct trends depending on the specific reaction mechanisms involved and the chemical nature of the antioxidant compounds present. This variability may be attributed both to the different analytical specificities of the assays employed and to synergistic or additive interactions among the various antioxidant compounds [48]. Among the analytical methods used, the CUPRAC assay yielded the highest overall antioxidant activity values. This method is based on the reduction of Cu (II) to Cu (I) under near-neutral pH conditions, which favour dissociation of phenolic hydroxyl groups and allow efficient evaluation of both hydrophilic antioxidants, such as flavonoids, and lipophilic antioxidants, including β-carotene and *α*-tocopherol [49,50]. Furthermore, the CUPRAC assay exhibits high selectivity and is minimally influenced by common juice constituents such as sugars and organic acids [49,51]. The CUPRAC assay revealed marked differences among the analysed beverages. The highest antioxidant activity was observed in the grapevine leaf-enriched grape juice (GJF), both immediately after production and after 30 days of refrigerated storage, with values of 22.20 ± 0.15 and 22.17 ± 0.18 mM TrE, respectively. GJ_0_ showed CUPRAC activity comparable to GJF, whereas GJ_30_ showed a lower value than GJ_0_. These observations are consistent with the higher concentrations of polyphenols, flavonoids, anthocyanins, carotenoids, and tocopherols measured in the investigated GJF preparation.

The ABTS assay confirmed the trend observed with CUPRAC, showing the highest antioxidant activity in fresh GJF (6.78 mM TrE), consistent with its elevated concentrations of polyphenols, flavonoids, anthocyanins, and lipophilic antioxidants (Table 1). Since the ABTS radical cation is soluble in both aqueous and organic media, this assay allows simultaneous determination of hydrophilic and lipophilic antioxidant compounds, including phenolics, carotenoids, and tocopherols [52]. Lower ABTS values were observed in the GJ samples (5.82 ± 0.09 and 5.05 ± 0.08 mM TrE for GJ_0_ and GJ_30_, respectively), whereas CGJ exhibited the lowest activity (2.62 ± 0.05 mM TrE), in agreement with its poorer phenolic profile. At t_30_, GJF showed a slightly lower ABTS value than at t_0_ (5.62 ± 0.09 vs. 6.78 ± 0.11 mM TrE, respectively).

In contrast, the DPPH assay generally provided lower estimates of antioxidant capacity compared with ABTS and CUPRAC, particularly in fresh samples (Table 1). At t_0_, GJF showed a lower DPPH value than GJ (0.88 ± 0.02 vs. 2.13 ± 0.05 mM TrE, respectively). At t_30_, GJF showed a higher DPPH value than at t_0_ (5.63 ± 0.10 vs. 0.88 ± 0.02 mM TrE, respectively), whereas GJ showed values of 1.85 ± 0.04 and 2.13 ± 0.05 mM TrE at t_30_ and t_0_, respectively.

The lower antioxidant values typically obtained by the DPPH assay are associated with the structural and chemical limitations of the DPPH radical itself. Since DPPH is generally dissolved in organic solvents, such as methanol or ethanol, hydrophilic antioxidant compounds may not be efficiently solubilized, thereby limiting their reactivity. Moreover, steric hindrance can significantly affect the accessibility of antioxidant molecules to the DPPH radical centre, resulting in slower reaction kinetics and lower apparent radical scavenging activity compared with the faster and more extensive reactions observed in ABTS and CUPRAC assays [52,53]. The higher DPPH scavenging activity observed in GJF at t_30_ than at t_0_ was not paralleled by the ABTS and CUPRAC assays, indicating an assay-dependent difference rather than a generalized increase in antioxidant capacity. Given the different reaction mechanisms, solvent environments, and sensitivities of antioxidant assays toward individual antioxidant compounds, this result should be interpreted cautiously. The chemical changes potentially responsible for the increased DPPH response were not specifically investigated in the present study; therefore, no specific mechanism can be established from the available data. Targeted chromatographic analyses would be required to identify the compounds potentially responsible for this behaviour. Overall, the antioxidant assays showed differences in antioxidant capacity between the investigated preparations, with the magnitude and direction of these differences depending on the analytical assay and experimental time point.

It should be acknowledged that the spectrophotometric assays used in the present study provide global indices of phenolic content and antioxidant capacity and may be affected by matrix components; therefore, they do not provide information on the identity and concentration of individual phenolic compounds. Targeted chromatographic analyses, such as HPLC-DAD or LC-MS/MS, would be required to characterize the individual phenolic compounds transferred from grapevine leaves to the fortified juice and to monitor their specific changes during storage.

These findings are consistent with the growing body of research on the valorization of grape-derived by-products as sources of phenolic and other bioactive compounds for food fortification. Recent studies and reviews have shown that the incorporation of grape by-products into food matrices can increase phenolic content and antioxidant capacity, although the magnitude of these effects depends on the type of by-product, extraction procedure, fortification level, and food matrix [54,55].

However, to the best of our knowledge, experimental studies specifically investigating the fortification of grape juice with grapevine leaf extract are currently lacking. Consequently, direct analytical comparisons with equivalent fortified beverages are not possible, and the present findings can only be contextualized against studies involving other grape-derived by-products or plant-based fortifying ingredients.

In this context, the present results extend this approach to grapevine leaves, showing that this comparatively underutilized grapevine by-product can contribute both hydrophilic and lipophilic antioxidant compounds to a grape-based beverage.

### 2.3. Tocol and Carotenoid Profile

The analysis of tocols and carotenoids further characterized the contribution of grapevine leaf extract to the phytochemical composition of the fortified juice (Table 2).

*α*-Tocopherol, *γ*-tocopherol, β-carotene, and lutein were exclusively detected in the enriched samples (GJF_0_ and GJF_30_), whereas they were not found in either GJ or CGJ. In particular, GJF_0_ contained 101.2 ± 2.1 µg/100 mL of *α*-tocopherol, 21.5 ± 0.6 µg/100 mL of *γ*-tocopherol, 61.1 ± 1.4 µg/100 mL of β-carotene, and 55.6 ± 1.2 µg/100 mL of lutein, confirming the contribution of grapevine leaf extract to the lipophilic antioxidant fraction of the fortified juice. At t_30_, lower concentrations of α-tocopherol (93.8 ± 1.9 µg/100 mL), β-carotene (36.9 ± 1.0 µg/100 mL), and lutein (46.5 ± 1.1 µg/100 mL) were observed than at t_0_, while measurable amounts remained detectable (Table 2). Tocopherols are well-known lipophilic antioxidants whose stability is strongly influenced by oxidative processes, light exposure, and storage temperature, whereas carotenoids are highly sensitive to heat, light, and oxidation, which generally leads to a progressive degradation during storage [56]. The magnitude of the differences observed between t_0_ and t_30_ was compound-dependent, with β-carotene showing the largest descriptive decrease, followed by lutein and α-tocopherol, whereas γ-tocopherol showed only limited variation. These observations suggest different retention patterns among the investigated lipophilic compounds under the conditions examined, although the present design does not allow a generalized storage effect to be established.

Nevertheless, despite the observed reductions, measurable amounts of these compounds remained after storage, indicating their partial retention under refrigerated conditions. Overall, these descriptive findings show that the investigated fortified preparation was characterized by higher concentrations of phenolic compounds and by the presence of carotenoids and tocopherols not detected in the non-fortified preparation, together with differences in antioxidant capacity depending on the analytical method and experimental time point.

### 2.4. Sensory Characterization of Grape Juices

**Figure 1:**
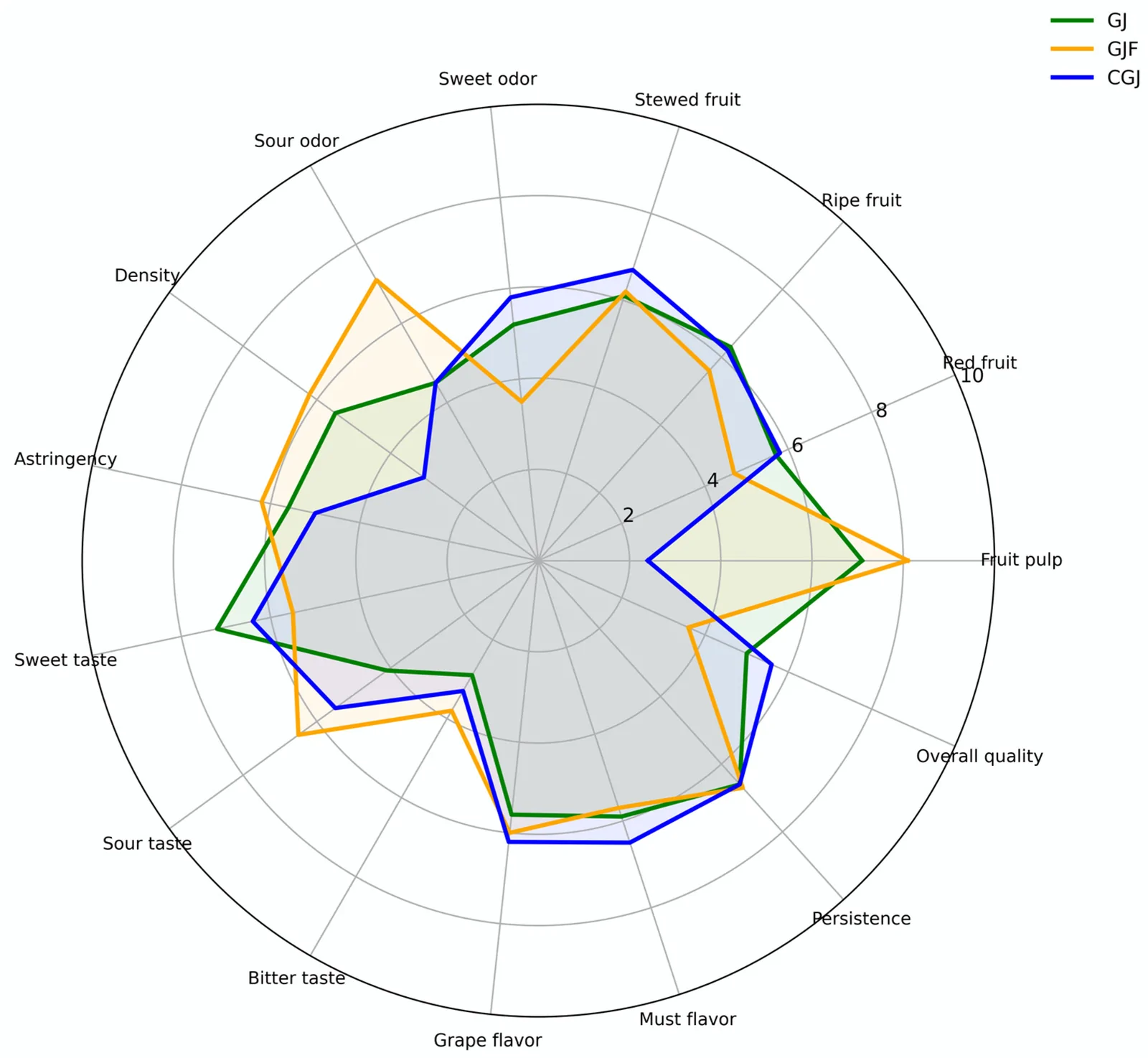
Radar chart displaying the sensory profiles of three grape-based beverages evaluated after 30 days of refrigerated storage at 4 °C: grape juice (GJ, green line), grape juice fortified with grapevine leaf extract (GJF, orange line), and commercial grape juice (CGJ, blue line), included as a commercial reference and analyzed at the same experimental time point. The chart presents the mean scores assigned by the trained sensory panel for 15 sensory attributes. Higher values indicate greater perceived intensity of the corresponding attribute.

After 30 days of refrigerated storage at 4 °C, the sensory profiles of the three grape juices differed substantially (Figure 1), with significant variations observed for several aroma, taste, and mouthfeel attributes (Table S1). Statistical evaluation of panel performance (Table S2) indicated generally non-significant replicate effects and limited interactions involving replicate evaluations, supporting satisfactory repeatability of the sensory assessments within the present panel. The assumptions underlying the parametric analysis were satisfied for most sensory attributes. Shapiro–Wilk testing indicated deviations from residual normality for astringency and bitter taste, whereas Levene’s test indicated heterogeneity of variance for ripe fruit odor. For these attributes, the product effect was additionally evaluated using the non-parametric Friedman test, with assessor treated as the blocking factor. Among all evaluated descriptors, fruit pulp particles represented the most discriminating sensory attribute, with significantly higher scores observed in GJ and GJF than in the commercial grape juice (CGJ).

The markedly lower score observed for CGJ may be attributed, at least in part, to the clarification and filtration processes typically applied during commercial juice production, which reduce the amount of suspended particulate matter. In contrast, the experimental juices (GJ and GJF) were not subjected to these technological treatments and therefore retained higher levels of pulp particles and colloidal material. Density also contributed significantly to sample discrimination, with GJF receiving the highest score, followed by GJ and CGJ. The lower perceived density of CGJ once again seems to be related to the effects of clarification and filtration, which generally result in a cleaner and less structured mouthfeel than minimally processed juices. GJF was characterized by greater perceived structural complexity and body than the non-fortified preparation.

The grape juice enriched with grapevine leaf extract (GJF) received the highest scores for sour odor, sour taste, bitterness, density, fruit pulp particles, and astringency, while obtaining the lowest scores for sweet odor, sweet taste, and overall sensory quality. The fortified preparation was characterized by greater acidity-, bitterness-, and mouthfeel-related attributes and lower sweetness perception. The opposite trends observed for sweet and sour odor were particularly noteworthy, as GJF exhibited the lowest sweet odor intensity and the highest sour odor intensity among all samples. This contrast in aromatic balance was characterized by stronger acidity-related aromatic notes and weaker sweetness-associated aroma perceptions in GJF than in the other investigated beverages.

The higher astringency perceived in GJF may be associated with its elevated concentration of phenolic compounds and flavonoids. Polyphenols are known to interact with salivary proteins, promoting protein aggregation and reducing oral lubrication, mechanisms widely recognized as the primary drivers of astringency perception [56,57]. Although previous studies have suggested that aroma compounds may modulate astringency through cross-modal interactions [58,59], the greater astringency observed in GJF is more plausibly attributed to its higher phenolic content than to specific volatile compounds.

Compared with GJF, both GJ and CGJ exhibited lower intensities of sourness, bitterness, and astringency. GJ showed the highest sweet taste intensity and intermediate values for most of the remaining sensory descriptors, resulting in an overall sensory quality score comparable to that of the commercial product. In contrast, CGJ displayed the most balanced sensory profile, combining relatively high sweet odor and sweet taste intensities with lower astringency and moderate acidity. Consequently, CGJ achieved the highest overall sensory quality score among the evaluated beverages, suggesting that an appropriate balance among sweetness, acidity, and mouthfeel attributes contributed to its higher overall sensory quality as assessed by the trained panel [60].

Overall, the fortified preparation showed a distinct sensory profile compared with the non-fortified grape juice, characterized by greater sourness, bitterness, and astringency and lower overall sensory quality. This represents an important limitation of the formulation at the fortification level investigated. Therefore, the present results do not indicate an unequivocal improvement in overall beverage quality but rather reveal a trade-off between phytochemical enrichment and sensory quality at the fortification level investigated. This finding emphasizes that increases in selected phytochemical and antioxidant indices should be evaluated together with their sensory consequences and highlights the need for further formulation optimization to achieve a more favorable balance between phytochemical enrichment and sensory quality [61,62].

This trade-off between phytochemical enrichment and sensory quality is consistent with previous studies on grape by-product fortification, in which increasing the incorporation of phenolic-rich grape-derived ingredients enhanced the phytochemical composition of the resulting foods but could also modify acidity, astringency, flavor, and overall sensory perception [63].

These observations emphasize that the technological feasibility of grape by-product valorization should be considered together with optimization of the fortification level to preserve sensory quality. Because only one fortification level was investigated, the present study cannot establish how reducing the amount of leaf extract to improve sensory quality would affect phytochemical enrichment or antioxidant-related indices. It therefore remains unknown whether a lower fortification level could preserve the compositional features associated with leaf incorporation while providing a more favorable sensory profile. Likewise, the present data cannot predict whether antioxidant-related indices at a lower fortification level would approach those observed in the commercial reference, which differs from the experimental juices in formulation and processing. Evaluation of multiple fortification levels will therefore be essential in subsequent formulation studies.

From a broader application perspective, some limitations related to the interpretation and potential use of the present findings should also be considered. The phytochemical enrichment and in vitro antioxidant capacity observed in the fortified juice should not be interpreted as evidence of physiological health benefits or of the safety of the formulation. Although grapevine leaves have a history of traditional use, the present study did not include toxicological or bioavailability assessments, and therefore the safety of the concentrated leaf extract at the investigated fortification level remains to be established. Moreover, higher concentrations of phenolic and other antioxidant compounds should not automatically be considered beneficial, since their biological effects depend on dose, bioavailability, metabolism, and interactions within the food matrix. In addition, pesticide residues and residual ethanol were not analytically quantified in the final formulation.

Moreover, because an ethanol-matched vehicle control was not included in the experimental design, a possible contribution of the small amount of ethanol used for extract reconstitution to some of the observed differences, particularly in the volatile and sensory profiles, cannot be completely excluded. Future studies should therefore include a vehicle control containing the same nominal ethanol concentration as GJF. Consequently, further studies should address toxicological safety, bioavailability, potential contaminants and pesticide residues, residual solvent content, and dose optimization. Furthermore, the regulatory status of grapevine leaf extract for the intended use would need to be established under the applicable food legislation, taking into account the extraction procedure, intended level and conditions of use, and documented history of food consumption. Therefore, the present formulation should be regarded as a proof-of-concept, and its suitability for food-scale or commercial application remains to be established.

### 2.5. Characterization of the VOCs Profile

The 30-day refrigerated period was selected as a predefined observation point for the preliminary characterization of the proof-of-concept formulation rather than as a formally established shelf-life or a factorial assessment of storage effects. Accordingly, the VOC profiles determined at t_30_ represent the characteristics of the investigated beverages specifically at this experimental time point and do not allow assessment of the temporal evolution of individual VOCs during refrigerated storage. Descriptive comparison of the investigated beverages showed differences in their volatile profiles at t_30_, including between GJ_30_ and GJF_30_.

However, because VOCs were not determined at t_0_, the present experimental design does not allow changes occurring during refrigerated storage to be evaluated or the immediate contribution of leaf extract incorporation to be distinguished from changes potentially occurring over the 30-day period. In addition, because an ethanol-matched vehicle control was not included, a possible contribution of the ethanol used for extract reconstitution to the observed VOC profile cannot be completely excluded.

Overall, 63 volatile compounds were detected and assigned in the headspace (HS) of the grape-based beverage samples: 5 esters, 12 aldehydes, 22 alcohols, 3 ketones, 10 terpenes, 7 acids, 3 furans, and 1 compound classified as other. Identification was based on complementary criteria, including MS library matching, comparison of experimental LRI values with literature data, and, when available, comparison with commercial standards. Compounds supported by commercial standards were considered confirmed under the applied analytical conditions, whereas those identified by MS and LRI comparison without a commercial standard were considered tentative. The identification criteria applied to each compound are reported in Table 3.

The analytical response of each VOC was expressed semi-quantitatively as relative peak area (RPA%), calculated according to Equation (1). As compound-specific calibration curves were not established, RPA% values represent relative analytical responses normalized to the internal standard and should not be interpreted as absolute VOC concentrations. Accordingly, these values are primarily intended for descriptive comparison of the response of the same VOC among the investigated beverages rather than for direct quantitative comparison among different VOCs.

(1) $$RPA\%=\frac{{VOC}_{peak\;area}}{{IS}_{peak\;area}}\cdot 100$$

Most of the detected VOCs have previously been reported in Aglianico wines and several grape-based juices from different cultivars [64,65,66,67,68,69,70,71,72].

The semi-quantitative HS-SPME/GC-MS profiles showed marked descriptive differences among the investigated beverages (Table 3). Alcohols generated prominent analytical responses in all three beverages. Among the detected alcohols, 1-hexanol showed a particularly high relative response in GJ_30_ and CGJ. In GJF_30_, ethanol, 1-hexanol, and cis-2-hexen-1-ol also showed comparatively high responses. These observations refer to semi-quantitative analytical responses and should not be interpreted as absolute or directly comparable concentrations among different VOCs.

Alcohols can originate from several biochemical and processing-related pathways and may contribute differently to the aroma profile depending on their identity and concentration [70]. C6 alcohols, including 1-hexanol and cis-2-hexen-1-ol, have previously been associated with vegetal notes in grape juices [65]. However, given the semi-quantitative nature of the present data, no direct inference regarding their individual sensory contribution can be made.

Terpenes also showed a distinctive response pattern in GJF_30_ compared with GJ_30_ and CGJ (Table 3). In particular, trans-geraniol and linalool showed substantially higher relative analytical responses in GJF_30_ than in the other investigated beverages. These compounds are among the monoterpenols reported in grapevine tissues and are commonly associated with floral aroma characteristics in grapes and wine [71].

Monoterpene alcohols have been reported to show an uneven distribution among grapevine organs and tissues. Previous studies have suggested that leaves may represent an important site of monoterpene occurrence and metabolism, while the distribution of these compounds and their glycosylated forms varies among leaves and berry tissues [71]. Geraniol, in particular, has been reported as a major monoterpene alcohol in leaves of different grape cultivars [71], whereas linalool has also been reported in grape berry tissues [71]. The comparatively high relative analytical responses of geraniol and other monoterpenes observed in GJF_30_ are therefore consistent with the incorporation of grapevine leaf extract; however, the present experimental design does not establish that the leaf extract was solely responsible for these differences. Because VOCs were determined only after 30 days of refrigerated storage and no ethanol-matched vehicle control was included, the respective contributions of leaf extract incorporation, the ethanol carrier, and changes occurring during storage cannot be separated.

### 2.6. Integrated Comparison of Volatile and Sensory Profiles

**Figure 2:**
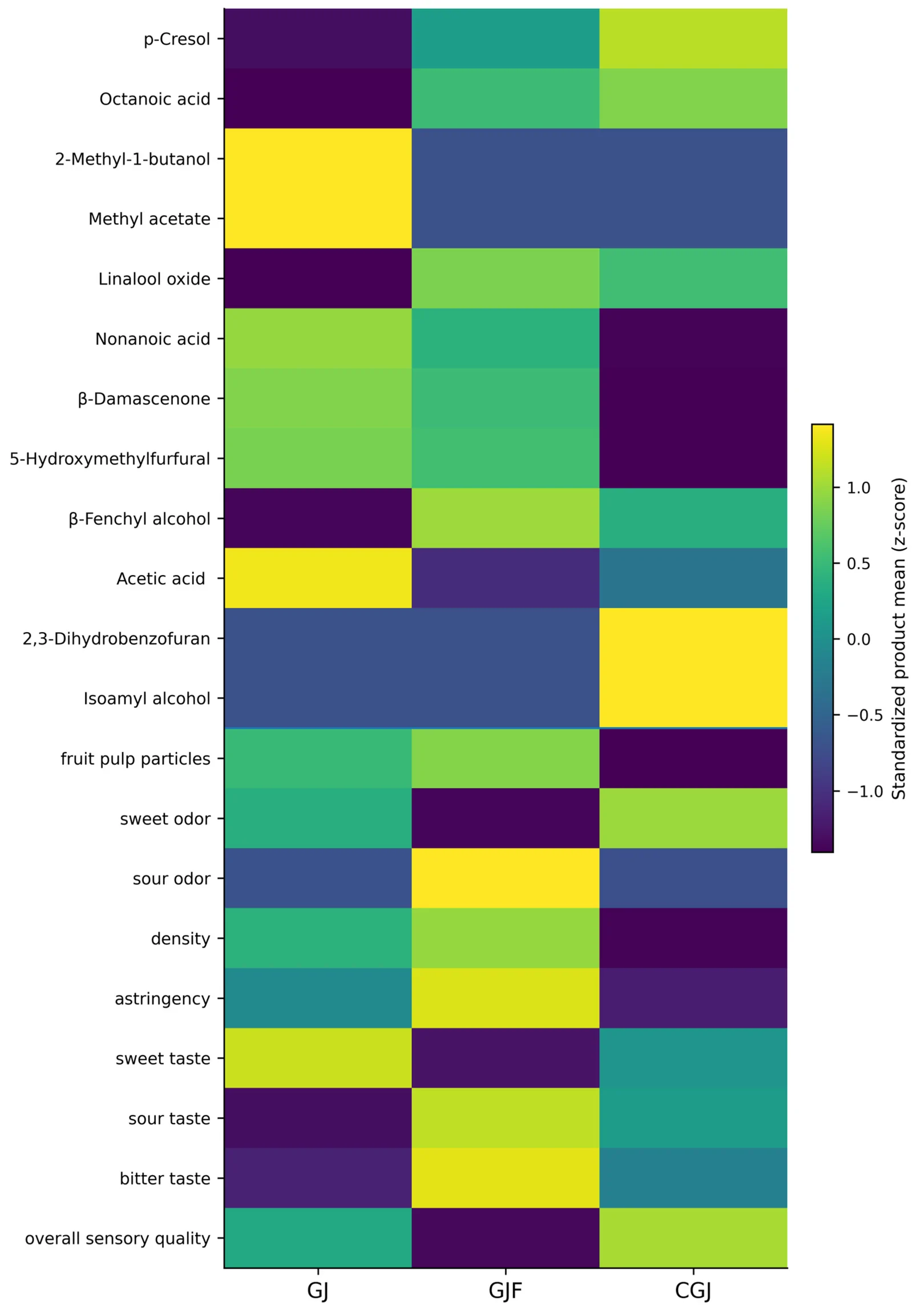
Integrated descriptive heatmap of the volatile and sensory profiles of grape juice (GJ), grape juice fortified with grapevine leaf extract (GJF), and commercial grape juice (CGJ). VOCs were selected using an exploratory PLS analysis based on Variable Importance in Projection (VIP) scores, whereas sensory attributes were selected according to significant product effects. Mean values for each variable were standardized across the three beverages to facilitate visualization of relative product profiles. The heatmap represents a descriptive product-level comparison and does not indicate statistical correlations between individual VOCs and sensory attributes.

To provide an integrated overview of the differences among the three beverages, VOCs identified by the exploratory PLS analysis as major contributors to sample discrimination (Table S3) were considered together with sensory attributes showing significant product effects. Although VOC and sensory measurements were linked at the bottle level, no replicate-level correlation analysis was retained because only nine bottle-level observations were available (3 beverages × 3 bottles), which was considered insufficient to support robust inference regarding individual VOC–sensory relationships. Instead, mean product values were standardized across GJ, GJF, and CGJ and jointly visualized in a descriptive heatmap (Figure 2). The integrated comparison revealed distinct product-level patterns in both volatile and sensory characteristics. GJF was characterized by relatively higher intensities of sour odor, density, astringency, sour taste, and bitter taste, together with lower sweet odor, sweet taste, and overall sensory quality. In contrast, GJ showed relatively greater sweet taste and lower sour and bitter taste, whereas CGJ was characterized by relatively higher sweet odor and overall sensory quality. The selected VOCs also displayed distinct product-dependent patterns. For example, β-fenchyl alcohol and linalool oxide were relatively more abundant in GJF, whereas 2-methyl-1-butanol, methyl acetate, acetic acid, β-damascenone, nonanoic acid, and 5-hydroxymethylfurfural showed different relative distributions among GJ, GJF, and CGJ (Figure 2). These patterns should be interpreted as a descriptive co-occurrence of volatile and sensory characteristics at the product level rather than as evidence of direct statistical or causal relationships between individual VOCs and sensory attributes. Overall, the integrated descriptive analysis showed that the investigated fortified preparation was characterized by a volatile and sensory profile distinct from those of the other investigated beverages. At the investigated fortification level, this profile included greater sourness, bitterness, and astringency and lower overall sensory quality, further emphasizing the need to optimize the formulation to balance phytochemical enrichment with sensory acceptability.

## 3. Materials and Methods

### 3.1. Grapevine Products

The grape juice and leaves used in this study were obtained from *Vitis vinifera* L. cv. Aglianico, a cultivar traditionally grown in southern Italy. The grape juice, supplied by the Mastroberardino winery (Atripalda, Italy), was free of skins, seeds, sulfur dioxide, and other oenological additives. Its physicochemical characteristics were as follows: pH 3.34, total sugars 258 g/L, total acidity 6.59 g/L (expressed as tartaric acid equivalents), and malic acid 0.95 g/L. Prior to use, the grape juice was pasteurized at 90 °C for 10 min.

The Aglianico leaves used in the present study were obtained from the same 2024 sampling campaign conducted in vineyards located in Taburno, Campania, southern Italy, as previously described and characterized in [35]. The vineyard was trained using a bilateral Guyot system and managed according to organic farming practices, with the farm exclusively cultivating grapes under an organic production system. Sampling was performed during the flowering/fruit-set period, coinciding with seasonal defoliation. Leaves were randomly collected from the lower sections of the shoots across different vineyard rows, consistent with the leaf-removal practice commonly performed at this phenological stage. After collection, damaged portions and foreign material were removed, and the leaves were thoroughly mixed to obtain a representative sample and promptly stored at −20 °C until processing. The leaves were subsequently freeze-dried using a Genesis 25SES freeze dryer (VirTis Co., Gardiner, NY, USA) and milled with a water-cooled grinder (IKA^®^-Werke GmbH & Co. KG, Staufen, Germany).

A total of 30 L of pasteurized Aglianico grape juice was used for the experimental beverage preparation. The homogeneous bulk juice was divided into two equal portions of 15 L each. One portion was maintained without leaf extract and used as the non-fortified reference beverage (GJ), whereas the second portion was fortified with grapevine leaf extract to obtain grape juice fortified with grapevine leaf extract (GJF).

The leaf extract was prepared by a single extraction using a leaf-to-solvent ratio of 1:10 (*w*/*v*). Accordingly, 75 g of freeze-dried leaf powder, corresponding to 5 g of freeze-dried leaves per litre of beverage to be fortified, was suspended in 750 mL of 80% (*v*/*v*) aqueous ethanol and allowed to macerate for 7 days at room temperature prior to filtration [73]. No further extraction of the residual leaf material was performed. No specific oxidation-control procedure, such as extraction under an inert atmosphere, was applied; however, maceration was performed in closed containers protected from light, thereby limiting exposure to the external environment.

After filtration, the 80% aqueous ethanol used as the extraction solvent was removed under reduced pressure using a Büchi Rotavapor R-300 (BÜCHI Labortechnik AG, Flawil, Switzerland). The resulting dried extract was reconstituted in 15 mL of absolute ethanol, corresponding to 1 mL per litre of beverage, and homogeneously incorporated into the 15 L portion of grape juice to obtain GJF. Thus, the ethanol used during extraction was removed before fortification, whereas the absolute ethanol used for reconstitution corresponded to a nominal addition of approximately 0.1% (*v*/*v*) to GJF. Residual ethanol was not analytically quantified in the final beverage. The small volume of ethanol used for reconstitution was selected to facilitate solubilization and homogeneous incorporation of constituents with different polarities present in the dried extract, particularly the less polar fraction, which would be less readily dispersed using water alone. The leaf extract was added after pasteurization to limit the thermal exposure of heat-sensitive leaf-derived constituents, particularly carotenoids and tocopherols. No additional thermal or specific decontamination treatment was applied to the extract before fortification. Microbiological stability of the beverages was subsequently assessed immediately after preparation (t_0_) and after 30 days of refrigerated storage (t_30_), as described in Section 3.2.

The gravimetric extraction yield was not determined because the entire extract obtained from the defined amount of leaf material was used for beverage fortification. Consequently, the fortification level is expressed on a leaf dry-weight equivalent basis (5 g of freeze-dried leaves per litre of grape juice) rather than as the mass concentration of the dried extract.

The selected fortification level (5 g freeze-dried leaf equivalent per litre of grape juice) was based on a recent characterization study conducted by our research group on Aglianico grapevine leaves, in which their phytochemical composition, including polyphenols, carotenoids, and tocols, and antioxidant capacity were comprehensively evaluated using chromatographic and spectrometric techniques [35]. Based on these compositional data, this amount was selected as a preliminary formulation level to provide an appreciable contribution of leaf-derived bioactive compounds, particularly carotenoids and tocols.

Following preparation, GJ and GJF were separately homogenized. At t_0_, three separate aliquots were independently withdrawn from each 15 L preparation for the initial analytical determinations. Thus, the t_0_ measurements represent technical/analytical replicates from the same beverage preparation rather than independently prepared beverage batches. Following t_0_ sampling, the remaining GJ and GJF were portioned into separate 750 mL dark-glass bottles and stored at 4 °C for 30 days. At t_30_, three different bottles of each beverage were independently opened and sampled for the corresponding analytical and sensory determinations. Thus, the t_30_ replicates represent independently stored subsamples originating from the same initial beverage preparation rather than independently prepared beverage batches or biological replicates.

For comparative purposes, a commercial grape juice (CGJ), produced by Folicello Società Agricola S.s. (Castelfranco Emilia, Italy), was used as a commercial reference sample. From the time of purchase until analysis, CGJ was maintained at 4 °C in its original unopened commercial packaging. The bottles were opened at the time of analysis, which was performed concurrently with the t_30_ analyses of GJ and GJF. CGJ was used as a commercial reference and was not subjected to the experimental t_0_–t_30_ storage protocol applied to the laboratory-prepared beverages.

### 3.2. Chemical Analysis

The following chemical analyses were performed using three replicate samples at production (t_0_) and after 30 days of storage at 4 °C (t_30_), according to the replication scheme described in Section 3.1 and Section 3.10. All reagents used in this study were purchased from Sigma-Aldrich (St. Louis, MO, USA). Selected phytochemical and antioxidant parameters were determined immediately after preparation (t_0_) and after 30 days of refrigerated storage at 4 °C (t_30_) to descriptively document changes observed within the investigated preparations over this predefined period. VOC profiling and sensory evaluation were performed only at t_30_ to characterize the beverages at that experimental time point. Microbiological analyses were performed at both t_0_ and t_30_. The t_0_–t_30_ observations were not treated as a factorial evaluation of storage and were not used to estimate a fortification × storage interaction.

### 3.3. Determination of Tocols

Tocols were extracted by alkaline saponification followed by solvent extraction, according to the method described by [74], using 0.1 mL of fruit juice as the starting material. The final extract was reconstituted in an n-hexane/isopropanol solution (99:1, *v*/*v*).

Chromatographic identification and quantification of tocols were carried out using a Dionex HPLC system equipped with an Ultimate 3000 pump and a Luna silica column (5 µm particle size, 250 × 4.6 mm i.d.; Phenomenex, Torrance, CA, USA). The mobile phase consisted of n-hexane, ethyl acetate, and acetic acid (97.3:1.8:0.9, *v*/*v*/*v*) delivered at a flow rate of 1.6 mL/min. Detection was performed with a Dionex RF 2000 fluorescence detector (Sunnyvale, CA, USA) set at an excitation wavelength of 290 nm and an emission wavelength of 330 nm.

Compound identification and quantification were achieved by comparison with authentic standards, as previously described by [75]. Chromatographic data were processed using Dionex Chromeleon software version 6.6 (Sunnyvale, CA, USA). Vitamin E activity was expressed as µg/100 mL tocopherol equivalents (TE), according to the procedure reported by [35].

### 3.4. Determination of Carotenoids

Carotenoids were extracted using the same saponification and solvent extraction protocol described for tocol analysis. The dried extract was reconstituted in an n-hexane/isopropanol solution (90:10, *v*/*v*). Carotenoid analysis was carried out by normal-phase HPLC using the chromatographic system described above. Separation was achieved using a multilinear gradient consisting of n-hexane and isopropanol, in which the concentration of isopropanol progressively increased from 10% to 20%, according to the method reported by [74].

Detection was performed spectrophotometrically at 450 nm. Carotenoids were identified based on their absorption spectra and by comparison with certified reference standards. Vitamin A activity was expressed as µg/100 mL retinol equivalents (REs), calculated according to the guidelines of the European Food Safety Authority [1].

### 3.5. Determination of Phenolic Compounds

Prior to analysis, grape juice samples were centrifuged at 11,000 rpm for 10 min using a laboratory centrifuge (Eppendorf, Hamburg, Germany). Total phenolic content (TPC) was determined using the Folin–Ciocalteu colorimetric assay, based on the reaction of phenolic compounds with the Folin–Ciocalteu reagent and subsequent spectrophotometric measurement, following the procedure previously described [76,77]. Quantification was performed using a gallic acid calibration curve, and the results were expressed as g GAE/L (Gallic Acid Equivalents). Total flavonoid content (TFC) was determined using the aluminium chloride colorimetric assay, based on the formation of complexes between aluminium ions and flavonoids, according to [78]. Quantification was performed using a rutin calibration curve, and the results were expressed as g Rutin Equivalents (RtE/L) [79]. Anthocyanin content was determined according to [77] and expressed as mg malvidin-3-glucoside equivalents (MGE/L).

### 3.6. Antioxidant Capacity

Antioxidant capacity (AC) was assessed using three complementary spectrophotometric assays: ABTS [2,2′-azinobis(3-ethylbenzothiazoline-6-sulfonic acid)], DPPH (2,2-diphenyl-1-picrylhydrazyl), and CUPRAC (cupric ion-reducing antioxidant capacity), following the procedures described by [35]. Briefly, suitably diluted samples were allowed to react with the corresponding assay reagents, and antioxidant capacity was evaluated from the resulting changes in absorbance. The ABTS and DPPH assays are based on the ability of antioxidants to quench the respective radical species, whereas the CUPRAC assay evaluates the reducing capacity of antioxidants toward Cu(II). Trolox was used as the calibration standard, and antioxidant capacity was expressed as mM Trolox equivalents (TrE).

### 3.7. Microbiological Analysis

The following microbiological analyses were performed on the beverages immediately after preparation (t_0_) and after 30 days of refrigerated storage at 4 °C (t_30_).

These analyses were performed according to the methodologies described by [80,81,82,83]. The following microbial groups were evaluated:

- Mesophilic aerobic bacteria (MAB), enumerated on Plate Count Agar (PCA) after incubation at 30 °C for 48 h;
- Psychrotrophic bacteria (PB), determined on PCA after incubation at 7 °C for 72 h;
- Lactic acid bacteria (LAB), analysed on de Man, Rogosa and Sharpe (MRS) agar incubated at 30 °C for 72 h;
- Acetic acid bacteria (AAB), quantified on Glucose-Yeast Extract-Calcium Carbonate (GYC) agar incubated at 30 °C for 5 days;
- Enterobacteriaceae (EB), determined on Violet Red Bile Glucose (VRBG) agar incubated at 37 °C for 72 h;
- Yeasts and moulds (Y&M), enumerated on Rose Bengal Chloramphenicol Agar (RBCA) incubated at 28 °C for 5 days.

The detection limit depended on the inoculation procedure and sample volume used. For mesophilic aerobic bacteria, psychrotrophic bacteria, lactic acid bacteria, and Enterobacteriaceae, 1 mL of undiluted sample was inoculated by the pour-plate method, corresponding to a theoretical detection limit of 1 CFU/mL. Acetic acid bacteria and yeasts and moulds were enumerated by spread plating 0.1 mL of undiluted sample, corresponding to a theoretical detection limit of 10 CFU/mL. Results with no detectable colonies were reported as below the corresponding detection limit.

All culture media and supplements were purchased from Merck KGaA (Darmstadt, Germany).

### 3.8. Analysis of Volatile Organic Compounds (VOCs)

After storing the beverages for 30 days at 4 °C, VOC profiles were determined by HS-SPME/GC-MS according to [84]. For this purpose, 5 mL of each beverage, 2 g of NaCl and 10 µL of 3-octanol (0.4 µg/mL), as an internal standard (IS), were put in a 20 mL headspace vial (HS). Each sample was incubated at 40 °C for 10 min.

An extraction temperature of 40 °C was selected as a compromise between promoting VOC release from the aqueous matrix into the headspace and maintaining favourable analyte partitioning onto the DVB/CAR/PDMS fibre coating [85].

Moderate extraction temperatures in this range have also been successfully applied in previous HS-SPME studies of grape- and wine-related matrices [86].

Volatiles were extracted for 50 min by HS-SPME using an MPS 2 autosampler (Gerstel, Mülheim, Germany) equipped with a DVB/CAR/PDMS (50/30 μm; 2 cm length; Supelco, Bellefonte, PA, USA) fibre. Desorption was automatically performed in the GC inlet at 250 °C for 10 min. The fibre was cleaned and conditioned daily before analysis.

GC-MS analysis was performed using an Agilent 7890A gas chromatograph (Agilent Technologies, Santa Clara, CA, USA) coupled with a 5975A mass spectrometer (Agilent Technologies, Santa Clara, CA, USA), equipped with a capillary HP-Innowax column (30 m × 0.25 mm × 0.5 µm). The carrier gas was helium (purity ≥ 99.999%) at a constant flow rate of 1.5 mL/min. The oven was programmed to start at an initial temperature of 40 °C, maintained for 1 min. The temperature was then increased to 60 °C at a rate of 2 °C/min, followed by a rise to 150 °C at 3 °C/min. Heating continued to 200 °C at 10 °C/min, and then to 240 °C at 25 °C/min, where it was held for 7 min.

The ion source and quadrupole were set at 230 °C and 150 °C, respectively. The mass detector operated in electron impact (EI) mode at 70 eV with a scan range of 30 to 300 *m*/*z*.

VOC identification was based on complementary criteria, including comparison of experimentally determined linear retention indices (LRIs) with literature values, comparison of acquired mass spectra (MS) with the NIST05/Wiley07 mass spectral libraries, and comparison of retention times and mass spectra with commercial reference standards (S), when available. The identification criteria applied to each individual VOC are reported in Table 3. Compounds identified using a commercial standard (S), together with the corresponding chromatographic and mass spectral information, were considered confirmed under the applied analytical conditions, whereas compounds assigned on the basis of MS library matching and LRI comparison without a commercial standard were considered tentatively identified. VOC data were expressed semi-quantitatively as relative peak area (RPA%), calculated as (VOC peak area/internal standard peak area) × 100. Commercial reference standards, when available, were used for compound identification and were not used to construct quantitative calibration curves. Because compound-specific calibration curves and response factors were not established, RPA% values represent relative analytical responses normalized to the internal standard and should not be interpreted as absolute VOC concentrations or as directly comparable concentrations among different VOCs. Individual VOC peak areas were obtained from the total ion chromatogram (TIC).

### 3.9. Sensory Analysis

After being stored for 30 days at 4 °C, the grape juice samples were evaluated by a trained panel of fourteen assessors (both sexes, aged 40–60 years). During the three weeks preceding the sensory evaluation, the assessors participated in 1-h training sessions. During these sessions, sensory descriptors and their definitions for grape juice odor, texture, and taste attributes were developed through brainstorming and panel consensus. Panelists were provided with a range of commercial grape juices, basic taste solutions, and other reference standards (Table S4) to establish common descriptive anchors and familiarize them with the use of the 9-point intensity scale [87].

Sensory analysis was conducted in three-day sessions, with panelists evaluating three samples per session. On the day of analysis, bottles were opened and grape juices were served at room temperature in 50 mL transparent plastic cups coded with a three-digit number. Natural water was provided to cleanse the palate between samples.

The panel assessed typical fruit odor traits, including red fruit, ripe fruit, stewed fruit and also sweet and acid/fermented odor notes. Among taste attributes, sweetness, sourness, bitterness and astringency were evaluated. Additionally, texture was described and analyzed in terms of density and presence of fruit pulp particles. Finally, the panel evaluated the must and grape flavor of the grape juices and the overall quality.

Evaluations were carried out in eight individual sensory booths under controlled environmental conditions (20 ± 2 °C; relative humidity, 50 ± 5%) and white illumination (850 lux). Sample serving sequences were balanced across assessors to minimize order effects, and responses were recorded on paper score sheets.

In accordance with the European General Data Protection Regulation (Regulation E.C. (2016) No 679/2016 [88] of the European Parliament and of the Council of 27 April 2016), all data were treated anonymously. The study protocol, including the sensory evaluation, received a favorable opinion from the Bioethics Committee of the University of Molise on 18 March 2026 (protocol no. 9874 of 4 March 2026). Written informed consent was obtained from all participants prior to their participation in the sensory evaluation.

### 3.10. Statistical Analysis

The nature of replication depended on the sampling time and analytical determination. For the laboratory-prepared beverages (GJ and GJF), determinations at t_0_ were performed on three separate aliquots independently withdrawn from the same homogeneous beverage preparation. Thus, at t_0_, *n* = 3 represents technical/analytical replicates and does not correspond to independent leaf extractions, beverage preparations, batches, or biological replicates. Following t_0_ sampling, GJ and GJF were portioned into separate 750 mL dark-glass bottles and stored at 4 °C for 30 days. At t_30_, three different bottles of each beverage were independently opened and sampled for analysis. Thus, the t_30_ replicates represent independently stored subsamples originating from the same initial beverage preparation rather than independently prepared beverage batches. The commercial grape juice (CGJ) was supplied in its original commercial packaging and stored under the same refrigerated conditions, with three separate bottles sampled at t_30_.

Results of instrumental analyses are expressed as mean ± standard deviation. Because GJ and GJF each originated from a single beverage preparation, the analytical replicates at t_0_ and the independently stored bottle-level samples at t_30_ were not treated as independent experimental replicates of the fortification treatment. Accordingly, inferential statistical comparisons among the laboratory-prepared beverages were not used to establish generalized treatment effects. Instrumental data are therefore interpreted descriptively, with the reported variability reflecting analytical variability at t_0_ and bottle-to-bottle variability at t_30_ within the investigated preparations.

For sensory analysis, the same three bottle-level samples of each beverage used for the instrumental determinations at t_30_ were evaluated by the trained panel. Each of the 14 panelists evaluated the sample originating from each of the three bottles of each beverage; therefore, the three sensory replicates corresponded to the three independently sampled bottles and were linked to the instrumental measurements at the bottle level. Sensory profiles were visualized using radar charts based on the mean intensity scores assigned by the trained panel to the evaluated sensory descriptors. The assumptions underlying parametric analysis of the sensory data were evaluated by assessing residual normality using the Shapiro–Wilk test and homogeneity of variances using Levene’s test. When these assumptions were not satisfied, the robustness of the product effect was additionally evaluated using the non-parametric Friedman test, with assessor treated as the blocking factor.

Partial least squares (PLS) analysis was applied to the VOC dataset as an exploratory multivariate approach to identify volatile compounds contributing most strongly to differentiation among the beverage profiles. VOCs with Variable Importance in Projection (VIP) scores > 1.0 were considered the main discriminating compounds. Although the VOC and sensory measurements were linked at the bottle level, replicate-level correlation analysis was not retained in the revised statistical approach because the dataset comprised only nine bottle-level observations (3 beverages × 3 bottles), which was considered insufficient to support robust inference regarding individual VOC–sensory relationships and could result in correlations driven primarily by between-beverage differences. Accordingly, integration of the VOC and sensory datasets was restricted to a descriptive product-level comparison. Product mean values for the selected VOCs and sensory attributes showing significant product effects were standardized across GJ, GJF, and CGJ as z-scores and jointly visualized as a heatmap. The integrated heatmap was therefore interpreted descriptively and not as evidence of statistical or causal relationships between individual VOCs and sensory attributes.

All statistical analyses and graphical representations were performed using Python (version 3.12.14; Python Software Foundation, Wilmington, DE, USA) with scikit-learn (version 1.7.2), SciPy (version 1.18.1), pandas (version 3.0.6), and Matplotlib (version 3.11.2) libraries.

## 4. Conclusions

Within the investigated proof-of-concept formulation, incorporation of *Vitis vinifera* leaf extract was associated with a phytochemically enriched profile and with differences in antioxidant and sensory characteristics compared with the non-fortified preparation. In particular, tocopherols and carotenoids were detected in the fortified preparation but not in the non-fortified samples. For some of these compounds, lower concentrations were observed at t_30_ than at t_0_, whereas appreciable amounts remained detectable after 30 days of refrigerated storage. These observations are descriptive and should not be interpreted as evidence of a generalized storage effect.

After 30 days of refrigerated storage, the fortified beverage was also characterized by a distinct volatile and sensory profile, including greater sourness, bitterness, and astringency and lower overall sensory quality. Because VOCs were determined only at t_30_, the present experimental design does not allow the immediate contribution of leaf extract incorporation to the volatile profile to be distinguished from changes potentially occurring during storage. Although VOC and sensory measurements were linked at the bottle level, their integrated comparison was restricted to a descriptive product-level analysis because only nine bottle-level observations were available (3 beverages × 3 bottles). Therefore, the co-occurrence of specific volatile and sensory characteristics should not be interpreted as evidence of direct statistical or causal relationships between individual VOCs and sensory attributes. Microbiological counts remained below the respective detection limits under the investigated conditions.

The observed phytochemical enrichment and in vitro antioxidant activity should not be interpreted as evidence of physiological health effects or as substantiation of specific health claims. Furthermore, the present study represents an exploratory proof-of-concept formulation and does not establish whether grapevine leaf extract is authorized for use in grape juice. Before any potential commercial application, its regulatory status and safety would need to be assessed according to the applicable food legislation, considering the extraction process, intended conditions of use, and documented history of food consumption.

Overall, the present findings provide preliminary evidence supporting the potential valorization of grapevine leaves as an ingredient for grape-based beverages, while also highlighting a trade-off between phytochemical enrichment and sensory quality at the fortification level investigated. Phytochemical enrichment was accompanied by less favorable sensory characteristics and therefore should not be interpreted as an overall improvement in beverage quality. Further formulation optimization is required to identify a more appropriate balance between phytochemical enrichment and sensory acceptability. Future studies should include independently prepared beverage batches, multiple fortification levels, and an ethanol-matched vehicle control to strengthen the experimental evidence. They should also assess formulation safety and regulatory compliance and evaluate chemical, microbiological, volatile, and sensory changes using a properly replicated longitudinal storage design.

## Figures and Tables

**Table 1:** Antioxidant Activity and Content of Total Polyphenols, Anthocyanins, and Flavonoids in the Analysed Grape Juice Samples.

Parameter	GJ_0_	GJF_0_	GJ_30_	GJF_30_	CGJ
**CUPRAC (mM TrE)**	21.91 ± 0.20	22.20 ± 0.15	3.09 ± 0.08	22.17 ± 0.18	1.79 ± 0.06
**DPPH (mM TrE)**	2.13 ± 0.05	0.88 ± 0.02	1.85 ± 0.04	5.63 ± 0.10	1.14 ± 0.03
**ABTS (mM TrE)**	5.82 ± 0.09	6.781 ± 0.11	5.05 ± 0.08	5.62 ± 0.09	2.62 ± 0.05
**Anthocyanins (mg MGE/L)**	65.10 ± 1.40	115.69 ± 2.35	69.67 ± 1.55	80.51 ± 1.95	93.55 ± 2.10
**Flavonoids (g RtE/L)**	3.07 ± 0.07	4.03 ± 0.09	2.89 ± 0.06	3.70 ± 0.08	1.73 ± 0.04
**Polyphenols (g GAE/L)**	7.14 ± 0.12	8.07 ± 0.13	6.31 ± 0.10	7.74 ± 0.11	3.97 ± 0.07

**GJ**, non-fortified Aglianico grape juice; **GJF**, Aglianico grape juice fortified with grapevine leaf extract; **CGJ**, commercial grape juice; **t_0_**, immediately after beverage preparation; **t_30_**, after 30 days of refrigerated storage at 4 °C; **CUPRAC**, cupric reducing antioxidant capacity; **DPPH**, 2,2-diphenyl-1-picrylhydrazyl radical scavenging assay; **ABTS**, 2,2′-azino-bis(3-ethylbenzothiazoline-6-sulfonic acid) radical cation scavenging assay. Data are expressed as mean ± standard deviation (*n* = 3). At t_0_, *n* = 3 represents three technical/analytical replicates obtained from separate aliquots of the same beverage preparation. At t_30_, *n* = 3 represents samples obtained from three independently stored bottles originating from the same initial beverage preparation. For CGJ, *n* = 3 represents three separate commercial bottles. Values are presented descriptively and were not used to infer generalized treatment effects or reproducibility across independently prepared beverage batches. **MGE**, malvidin-3-glucoside equivalents; **RtE**, rutin equivalents; **GAE**, gallic acid equivalents; **TrE**, Trolox equivalents.

**Table 2:** Tocol and Carotenoid Profiles of the Analysed Grape Juice Samples.

Compounds	GJ_0_	GJF_0_	GJ_30_	GJF_30_	CGJ
*α*-tocopherol	ND	101.2 ± 2.1	ND	93.8 ± 1.9	ND
*γ*-tocopherol	ND	21.5 ± 0.6	ND	20.3 ± 0.5	ND
TE (µg/100 mL)	ND	101.4 ± 2.2	ND	94.0 ± 2.0	ND
*β*-carotene	ND	61.1 ± 1.4	ND	36.9 ± 1.0	ND
Lutein	ND	55.6 ± 1.2	ND	46.5 ± 1.1	ND
RE (µg/100 mL)	ND	10.2 ± 0.3	ND	6.2 ± 0.2	ND

**GJ**, non-fortified Aglianico grape juice; **GJF**, Aglianico grape juice fortified with grapevine leaf extract; **CGJ**, commercial grape juice; **t_0_**, immediately after beverage preparation; **t_30_**, after 30 days of refrigerated storage at 4 °C; **ND**, not detected; **TE**, Tocopherol Equivalents; **RE**, Retinol Equivalents. Data are expressed as mean ± standard deviation (*n* = 3). At t_0_, *n* = 3 represents three technical/analytical replicates obtained from separate aliquots of the same beverage preparation. At t_30_, *n* = 3 represents samples obtained from three independently stored bottles originating from the same initial beverage preparation. For CGJ, *n* = 3 represents three separate commercial bottles. Values are presented descriptively and were not used to infer generalized treatment effects or reproducibility across independently prepared beverage batches.

**Table 3:** Volatile organic compounds (VOCs) detected by HS-SPME/GC-MS in non-fortified Aglianico grape juice (GJ_30_), grape juice fortified with grapevine leaf extract (GJF_30_), and commercial grape juice (CGJ), analyzed after 30 days of refrigerated storage.

Volatile Compounds	Code	RIsp/ LRIt	ID	GJ_30_	GJF_30_	CGJ
**Esters**						
Methyl acetate	E1	845/844	LRI/MS/S	16.7 ± 1.5	ND	ND
Ethyl acetate	E2	899/900	LRI/MS/S	261.3 ± 4.2	354.3 ± 21.0	66.8 ± 7.2
Isoamyl acetate	E3	1114/1114	LRI/MS/S	ND	ND	9.0 ± 0.8
Ethyl hexanoate	E4	1244/1240	LRI/MS/S	5.8 ± 0.8	ND	5.7 ± 0.4
Hexyl acetate	E5	1269/1269	LRI/MS/S	5.1 ± 0.7	32.3 ± 3.9	8.8 ± 0.3
**Aldehydes**						
2-Methylbutanal	Ald1	919/922	LRI/MS/S	23.0 ± 2.1	34.6 ± 3.1	ND
3-Methylbutanal	Ald2	920/924	LRI/MS/S	25.9 ± 1.2	47.3 ± 9.2	ND
Hexanal	Ald3	1084/1084	LRI/MS/S	50.2 ± 3.5	110.1 ± 9.7	7.1 ± 0.9
2-Hexenal	Ald4	1242/1240	LRI/MS/S	119.5 ± 9.5	1705.8 ± 212.9	ND
Octanal	Ald5	1286/1284	LRI/MS/S	0.4 ± 0.1	0.5 ± 0.1	0.1 ± 0.0
2-Heptenal	Ald6	1341/1342	LRI/MS/S	0.2 ± 0.4	0.8 ± 0.1	ND
Nonanal	Ald7	1404/1404	LRI/MS/S	23.8 ± 1.5	238.6 ± 12.6	83.7 ± 3.3
2,4-Heptadienal	Ald8	1470/1472	LRI/MS/S	ND	ND	1.4 ± 0.1
Furfural	Ald9	1473/1476	LRI/MS/S	105.6 ± 2.1	762.4 ± 36.6	348.1 ± 17.2
Decanal	Ald10	1510/1511	LRI/MS/S	8.3 ± 1.1	ND	10.6 ± 0.9
Benzenacetaldehyde	Ald11	1651/1649	LRI/MS/S	20.2 ± 3.8	134.3 ± 1.2	10.6 ± 0.4
5-Hydroxymethylfurfural	Ald12	2428/2429	LRI/MS/S	79.4 ± 2.0	69.1 ± 2.4	ND
**Alcohols**						
Ethanol	Alc1	934/935	LRI/MS/S	1677.7 ± 50.0	2698.8 ± 48.1	2864.7 ± 92.9
2-Methyl-2-butanol	Alc2	1003/1006	LRI/MS/S	10.8 ± 1.6	109.6 ± 5.0	ND
1-Propanol	Alc3	1030/1033	LRI/MS/S	8.4 ± 1.3	502.1 ± 26.6	3.3 ± 0.1
Isobutyl alcohol	Alc4	1105/1106	LRI/MS/S	47.6 ± 2.3	113.1 ± 6.7	31.8 ± 3.1
Isoamyl alcohol	Alc5	1228/1228	LRI/MS/S	ND	ND	360.6 ± 11.1
2-Methyl-1-butanol	Alc6	1229/1231	LRI/MS/S	236.1 ± 20.5	ND	ND
1-Pentanol	Alc7	1253/1254	LRI/MS/S	ND	119.4 ± 4.1	6.2 ± 0.5
2-Heptanol	Alc8	1326/1328	LRI/MS/S	32.9 ± 2.7	78.5 ± 15.8	4.1 ± 0.8
1-Hexanol	Alc9	1365/1365	LRI/MS/S	2875.4 ± 30.9	2689.3 ± 41.5	2365.4 ± 83.6
*trans*-3-Hexen-1-ol	Alc10	1398/1398	LRI/MS/S	82.4 ± 6.3	234.6 ± 4.4	22.4 ± 1.0
*cis*-3-Hexen-1-ol	Alc11	1400/1401	LRI/MS/S	212.5 ± 17.4	1141.7 ± 89.2	18.1 ± 0.7
*cis*-2-Hexen-1-ol	Alc12	1409/1410	LRI/MS/S	789.2 ± 53.8	2074.7 ± 36.9	171.9 ± 13.6
*trans*-2-Hexen-1-ol	Alc13	1416/1415	LRI/MS/S	27.3 ± 3.1	102.5 ± 7.0	9.3 ± 1.6
1-Octen-3-ol	Alc14	1451/1426	LRI/MS/S	47.1 ± 3.2	249.5 ± 18.1	21.7 ± 1.9
1-Heptanol	Alc15	1464/1465	LRI/MS/S	30.1 ± 2.0	128.4 ± 1.3	15.8 ± 1.2
2-Ethylhexanol	Alc16	1486/1484	LRI/MS/S	8.3 ± 0.4	85.9 ± 1.8	26.4 ± 1.9
1-Octanol	Alc17	1561/1561	LRI/MS/S	25.8 ± 0.8	431.2 ± 3.9	38.9 ± 1.7
*trans*-2-Octen-1-ol	Alc18	1644/1645	LRI/MS/S	10.5 ± 2.1	162.1 ± 4.1	6.2 ± 1.1
*β*-Fenchyl alcohol	Alc19	1680/1679	LRI/MS/S	83.5 ± 8.1	463.2 ± 12.8	361.8 ± 15.9
1-Nonanol	Alc20	1650/1656	LRI/MS/S	0.2 ± 0.1	1.3 ± 0.1	0.2 ± 0.0
Benzenemethanol	Alc21	1864/1865	LRI/MS/S	0.8 ± 0.1	0.4 ± 0.1	1.2 ± 0.1
Benzeneethanol	Alc22	1882/1880	LRI/MS/S	157.7 ± 10.4	559.4 ± 18.6	142.6 ± 10.5
**Ketones**						
4-Methyl-2-heptanone	K1	1212/1211	LRI/MS/S	14.5 ± 0.9	83.7 ± 5.8	ND
Acetoin	K2	1288/1290	LRI/MS/S	8.8 ± 0.6	33.9 ± 2.1	5.0 ± 0.5
6-Methyl-5-hepten-2-one	K3	1338/1338	LRI/MS/S	7.6 ± 1.1	171.4 ± 1.3	5.5 ± 0.6
**Terpenes**						
Linalool oxide	T1	1467/1467	LRI/MS/S	14.5 ± 0.4	92.7 ± 0.7	82.2 ± 0.8
Nerol oxide	T2	1488/1469	LRI/MS/S	ND	ND	45.6 ± 6.1
Vitispirane	T3	1519/1515	LRI/MS	12.4 ± 1.9	77.2 ± 9.7	54.1 ± 13.5
Linalool	T4	1553/1557	LRI/MS/S	187.2 ± 10.1	1668.8 ± 12.8	47.8 ± 2.1
Hotrienol	T5	1619/1620	LRI/MS/S	22.5 ± 10	192.4 ± 11.6	25.1 ± 2.8
4-Terpineol	T6	1605/1607	LRI/MS/S	0.5 ± 0.1	1.8 ± 0.1	ND
p-Cresol	T7	2088/2087	LRI/MS/S	35.9 ± 3.2	101.7 ± 2.4	143.6 ± 10.2
*ß*-Citronellol	T8	1770/1768	LRI/MS/S	5.5 ± 1.1	215.7 ± 7.6	ND
*β*-Damascenone	T9	1842/1838	LRI/MS/S	29.7 ± 0.9	25.1 ± 2.6	ND
*trans*-Geraniol	T10	2045/2045	LRI/MS/S	19.5 ± 4.5	6171.2 ± 35.2	ND
**Acids**						
Acetic acid	A1	1467/1467	LRI/MS/S	146.7 ± 4.6	92.8 ± 1.2	109.0 ± 6.8
Hexanoic acid	A2	1848/1848	LRI/MS/S	52.1 ± 3.4	442.4 ± 14.6	68.1 ± 6.5
Heptanoic acid	A3	1952/1952	LRI/MS/S	2.7 ± 0.4	2.3 ± 0.1	2.2 ± 0.1
*trans*-2-Hexenoic acid	A4	1994/1990	LRI/MS	32.4 ± 2.1	218.7 ± 12.1	ND
Octanoic acid	A5	2073/2074	LRI/MS/S	9.8 ± 1.5	89.0 ± 1.2	103.6 ± 3.0
**Furans**						
2-Acetylfuran	F1	1514/1512	LRI/MS/S	8.8 ± 0.4	32.1 ± 0.8	11.5 ± 0.6
2,3-Dihydrobenzofuran	F2	2399/2389	LRI/MS	ND	ND	6.3 ± 0.1
2,2-dimethyl-5-(1-methyl-1-propenyl)-tetrahydrofuran	F3	1235/1231	LRI/MS	ND	ND	6.2 ± 0.2
**Others**						
2,6,6-Trimethyl-2-vinyltetrahydropyran	O1	1095/1096	LRI/MS	ND	16.8 ± 0.5	50.2 ± 2.6

**GJ_30_**, non-fortified Aglianico grape juice after 30 days of refrigerated storage at 4 °C; **GJF_30_**, Aglianico grape juice fortified with grapevine leaf extract after 30 days of refrigerated storage at 4 °C; **CGJ**, commercial grape juice analyzed at the same experimental time point. Data are expressed as semi-quantitative relative peak area (RPA%) and reported as mean ± standard deviation (*n* = 3). RPA% values represent relative analytical responses normalized to the internal standard and do not represent absolute VOC concentrations. Because compound-specific response factors were not established, values for different VOCs should not be interpreted as directly comparable concentrations. For GJ_30_ and GJF_30_, *n* = 3 represents samples obtained from three independently stored bottles originating from the same initial beverage preparation. For CGJ, *n* = 3 represents three separate commercial bottles. Values are presented descriptively and were not used to infer generalized treatment effects or reproducibility across independently prepared beverage batches. **LRIsp**, linear retention index relative to n-alkanes (C8–C20) on an HP-Innowax capillary column; **LRIt**, linear retention index values reported in the literature for an equivalent capillary column; **ID**, identification method; **LRI**, linear retention index; **MS**, mass spectrum; **S**, commercial standard; **ND**, not detected.

## Data Availability

The original contributions presented in this study are included in the article/Supplementary Material. Further inquiries can be directed to the corresponding authors.

## Supplementary Materials

The following supporting information can be downloaded at: https://www.mdpi.com/article/10.3390/molecules31193502/s1, Table S1. Sensory attributes (mean values ± SD) of GJ, GJF and CGJ grape juices. Different letters within each line indicate statistically significant differences between groups (*p* < 0.05), as determined by one-way ANOVA followed by Tukey’s HSD post hoc test (*** *p* < 0.001; ** *p* < 0.01; * *p* < 0.05). Table S2. Evaluation of Panel Performance as reported by 3-way ANOVA performed with Panel Check. The interaction Assessor*Product measures the consistency of individual assessors across different products. It indicates whether panelists are using the rating scales differently for specific products. If the *p* value is not significant it means that the panel is in consensus: panel members perceive the product differences similarly. The interaction Product*Replicate is not significant for all the attributes. This interaction measures whether the assessors’ evaluation of a specific product changed from the first time they tasted/tested it to a subsequent session (replication). A not significant *p*-value suggests that the panel evaluates the products the same way across all replicates. The interaction Assessor*Replicate has a not significant *p*-value for all the attributes. This means that the panel have the same grade mean for all the products. Table S3. Variable Importance in Projection (VIP) scores obtained from the PLS model used for VOC selection. Table S4. Sensory attributes of grape juices reported on the evaluation sheet, their definitions and references. The number in brackets indicates the intensity scale value.

## Abbreviations

The following abbreviations are used in this manuscript:

**Abbreviation**	**Full Term**
ABTS	2,2′-Azinobis (3-ethylbenzothiazoline-6-sulfonic acid)
CGJ	Commercial Grape Juice
CUPRAC	Cupric Ion Reducing Antioxidant Capacity
DPPH	2,2-Diphenyl-1-picrylhydrazyl
GAE	Gallic Acid Equivalents
GC-MS	Gas Chromatography–Mass Spectrometry
GJ	Grape Juice
GJF	Grape Juice Fortified with *Vitis vinifera* Leaf Extract
HS-SPME	Headspace Solid-Phase Microextraction
LRI	Linear Retention Index
MGE	Malvidin-3-glucoside Equivalent
PLS	Partial Least Squares
RE	Retinol Equivalent
RPA	Relative Peak Area
RtE	Rutin Equivalent
TE	Tocopherol Equivalent
TrE	Trolox Equivalent
VIP	Variable Importance in Projection
VOCs	Volatile Organic Compounds
IS	Internal Standard
