# Supplementary material for: Exploratory Characterization of Grape Juice Fortified with Vitis vinifera Leaf Extract: A Proof-of-Concept Study
Source: Molecules. 2026 Oct 1;31(19):3502. doi: 10.3390/molecules31193502 (PMC13666728; doi:10.3390/molecules31193502)
Supplement: Supplementary file 1 [file molecules-31-03502-s001.zip › molecules-4574123-supplementary.pdf]

Table S1. Sensory attributes (mean values ± SD) of GJ, GJF, and CGJ grape juices. Different letters within the same row indicate significant differences among beverages according to one-way ANOVA followed by Tukey's HSD post hoc test. Significance levels: *** p < 0.001; ** p < 0.01; * p < 0.05; ns, not significant. For sensory attributes not satisfying parametric assumptions, the product effect was additionally evaluated using the Friedman test, as described in the main manuscript.

Sensory attributes	GJ	GJF	CGJ	*p*
Fruit pulp particles	7,1 ± 0,2[b]	8,1 ± 0.3[c]	2,4 ± 0,3[a]	***
Red fruit odor	5,7 ± 0,3[a]	4,7 ± 0.4[b]	5,8 ± 0.2[a]	**
Ripe fruit odor	6,3 ± 0.5[a]	5,6 ± 0.3[a]	6,2 ± 0,3[a]	ns
Stewed fruit odor	6,1 ± 0.3[a]	6,2± 0.5[a]	6,7 ± 0.1[a]	ns
Sweet odor	5,2± 0.4[b]	3,5 ± 0,2[a]	5,8 ± 0,4[b]	***
Sour odor	4,5 ± 0,6[a]	7,1 ± 0,4[b]	4,5 ± 0,6[a]	***
Density	5,5 ± 0,3[b]	6,2 ± 0,2[c]	3,1 ± 0,3[a]	***
Astringency	5,6 ± 0.6[ab]	6,2 ± 0,2[b]	5,0 ± 0,2[a]	**
Sweet taste	7,2 ± 0,2[b]	5,5 ± 0,4[a]	6,4 ± 0,3[b]	***
Sour taste	4,1 ± 0,1[a]	6,5 ± 0,2[c]	5,5 ± 0,4[b]	***
Bitter taste	2,9 ± 0,1[a]	3,8 ± 0,2[c]	3,3 ± 0,1[b]	**
Grape flavor	5,6 ± 0,2[a]	6,0 ± 0,4[a]	6,2 ± 0,3[a]	ns
Must flavor	5,9 ± 0,3[a]	5,7 ± 0,3[a]	6,5 ± 0,3[a]	ns
Persistence	6,6± 0,3[a]	6,7 ± 0,4[a]	6,6 ± 0,4[a]	ns
Overall sensory quality	5,0 ± 0,5[b]	3,6 ± 0.4[a]	5,6 ± 0.3[b]	***

Table S2. Evaluation of panel performance by three-way ANOVA using PanelCheck.
The Product × Replicate interaction was non-significant for most sensory attributes, although significant interactions were observed for sour odor, persistence, and overall sensory quality.

	Fruit pulp particles	Red fruit odor	Ripe fruit odor	Stewed fruit odor	Sweet odor	Sour odor	Density	Astringency	Sweet taste	Sour taste	Bitter taste	Grape Flavor	Must Flavor	Persistence	Overall sensory quality
GJF	8.071	4.714	5.643	6.238	3.452	7.095	6.214	6.238	5.452	6.476	3.833	5.952	5.738	6.667	3.571
CGJ	2.405	5.810	6.238	6.667	5.833	4.452	3.143	5.048	6.381	5.524	3.262	6.214	6.452	6.571	5.619
GJ	7.095	5.738	6.286	6.143	5.214	4.476	5.476	5.595	7.167	4.119	2.881	5.643	5.905	6.595	4.976
Assessor Effect (F value)	1.56	3.50	1.82	3.63	2.28	2.60	3.81	2.59	6.71	12.12	21.45	3.06	5.26	1.89	1.71
p-values	0.163	0.003	0.087	0.003	0.037	0.014	0.002	0.019	0.000	0.000	0.000	0.008	0.000	0.082	0.116
Product Effect (F value)	109.21	2.57	0.74	1.13	12.41	13.35	32.19	4.06	13.15	32.52	6.88	0.57	1.41	0.02	5.08
p-values	0.000	0.096	0.492	0.337	0.000	0.001	0.000	0.069	0.003	0.000	0.004	0.577	0.263	0.975	0.016
Replicate Effect (F value)	2.44	1.75	0.10	1.27	1.86	0.78	0.73	0.26	0.78	0.33	0.007	0.35	3.42	0.08	0.81
p-values	0.164	0.197	0.908	0.331	0.184	0.479	0.499	0.778	0.489	0.731	1.000	0.706	0.059	0.924	0.457
Assessor x Product Interaction (F value)	1.56	5.87	5.57	1.78	4.05	4.09	3.29	1.61	1.85	1.60	1.02	4.86	3.07	2.85	7.32
p-values	0.085	0.000	0.000	0.039	0.000	0.000	0.000	0.072	0.029	0.076	0.465	0.000	0.000	0.001	0.000
Product x Replicate Interaction (F value)	0.45	0.99	2.09	0.74	0.77	3.15	0.94	1.21	1.29	0.91	0.33	1.50	0.83	3.24	2.56
p-values	0.773	0.422	0.095	0.570	0.553	0.022	0.448	0.320	0.285	0.476	0.854	0.215	0.512	0.019	0.049
Assessor x Replicate Interaction (F value)	0.78	0.74	1.68	1.66	1.15	2.05	1.16	0.71	1.48	0.88	0.59	1.06	1.21	0.82	1.32
p-values	0.755	0.791	0.056	0.061	0.327	0.014	0.316	0.824	0.112	0.629	0.928	0.416	0.277	0.703	0.196
LSD	0.84	1.11	1.23	0.76	1.02	1.28	0.82	0.99	0.77	0.60	0.53	1.12	0.92	1.01	1.37

**Bonferroni LSD**	1.05	1.38	1.55	0.95	1.27	1.63	1.02	1.32	1.01	0.75	0.66	1.41	1.14	1.31	1.71

Table S3. Variable Importance in Projection (VIP) scores obtained from the exploratory PLS analysis of volatile organic compounds (VOCs).
VOCs with VIP scores > 1.0 were considered relevant contributors to sample discrimination in the exploratory PLS analysis.

VOC	VIP score
Octanoic acid	1.454
2-Methyl-1-butanol	1.432
p-Cresol	1.425
Methyl acetate	1.421
Linalool oxide	1.405
β-Fenchyl alcohol	1.347
Acetic acid	1.312
β-Damascenone	1.235
Nonanoic acid	1.222
Vitispirane	1.210
5-Hydroxymethylfurfural	1.205
Furfural	1.132
Decanoic acid	1.119
Isoamyl alcohol	1.119
2,2-dimethyl-5-(1-methyl-1-propenyl)-tetrahydrofuran	1.119
2,3-Dihydrobenzofuran	1.118
Isoamyl acetate	1.117
2,4-Heptadienal	1.116
Nerol oxide	1.115
Nonanal	1.050
2-Ethylhexanol	1.031

Table S4. Sensory descriptors, definitions, and reference standards used during panel training. Values in parentheses indicate the corresponding points on the 9-point intensity scale.

Sensory Attribute	Definition	Reference standard
**Appearance**		
Fruit pulp particles	Perceived amount of suspended or visible fruit pulp particles in the juice.	None (1); Noticeable (9)
**Aroma**		
Red fruit odor	Evaluating intensity of characteristic odor of red fruit: notes of strawberry, raspberry, red currant, cherry and blackcurrant.	Distilled water (1); berry purée prepared from thawed berries immediately before each panel session (9)
Ripe fruit odor	Evaluating intensity of characteristic odor of ripe fruit	Distilled water (1); grape purée prepared from ripe grapes immediately before each panel session (9).
Stewed fruit odor	Measuring intensity of characteristic odor of cooked or jammy fruit.	Distilled water (1); dried plum purée (9)
Sweet odor	Measuring intensity of characteristic odor of sugar, fruits or caramel	Distilled water (1); 1 g vanillin in 500 mL distilled water (9)
Sour odor	Measuring intensity of characteristic acidic or fermented odor	Distilled water (1); red wine vinegar (9)
**Mouthfeel - Taste**		
Density	Perceived thickness, body, or fullness of the juice in the mouth	Water (1); clear apple juice (5); pear juice (9)

Astringency	Drying, puckering, and rough sensation perceived in the mouth.	Distilled water (1); tannic acid solution 1g/L (9)
Sweet taste	Measuring intensity of the sugar specific sensation	1.0% sucrose solution (1)<br>5.0% sucrose solution (9)
Sour taste	Measuring intensity of the sensation caused by acidic substances	0.05% citric acid solution (1)<br>0.08% citric acid solution (9)
Bitter taste	Measuring intensity of the sensation caused by bitter substances	0,004% caffeine solution (1)<br>0.01% caffeine solution (9)
Grape flavor	Intensity of the typical grape aroma perceived retronasally	Distilled water (1); grape purée prepared from ripe grapes immediately before each panel session (9)
Must flavor	Intensity of the typical must aroma perceived retronasally	Distilled water (1); Cooked wine (9)
Persistence	Intensity of retronasally perceived aromas 1 min after swallowing	None (1); Strong (9)
Overall sensory quality	Overall assessment of the sensory quality of the sample considering its appearance, aroma, taste, and mouthfeel characteristics.	Very low overall sensory quality (1); Very high overall sensory quality (9).
